# Cholera: A deadly but unnoticed outbreak in Pakistan

**DOI:** 10.1016/j.amsu.2022.104179

**Published:** 2022-07-14

**Authors:** Rida Siddiqui

**Affiliations:** Karachi Medical and Dental College, Pakistan

Any new outbreak or emergence of diseases can substantially challenge the already vulnerable healthcare system of Pakistan. This was seen during the pandemic of COVID19.Within just few years of the pandemic, the rising cases of Cholera in Pakistan especially in cities of Sindh and Baluchistan are of great concern.

Cholera is an infectious disease of the intestines caused by vibrio cholera resulting in severe vomiting, diarrhea, circulatory shock and even collapse. It has a fatality rate of 25–50% [1]. One of the major causes of Cholera spread is the inaccessibility to clean water resources, proper sanitation conditions and most susceptible group of people are the ones living in slums as WHO remarked [2].

One of the very first reported case of cholera was from Pir Koh, a town in Baluchistan on April 17.Since then, 2000 people have reported with infection while 6 have been dead as reported by CNN [3]. As of April 2022,129 cases have been reported from 6 public and private hospitals in Karachi [4] Most these cases are due to lack of clean water, poor sanitation and hand washing practices and negligence to personal hygiene along with some environmental and seasonal factors such as hot air temperatures and heavy rainfall [5].

91% of Karachi's water supply is unfit for drinking purposes as is contaminated with samples of Shigella, E coli and Cholera [6] Contaminated and unfit drinking water substantially increases the risk of water borne diseases and spread in a population.

There is a need of setting up proper sewage waste management plants, alongside educating masses about healthy sanitation habits in Pakistan. Creating awareness and launching campaigns for infectious diseases in Pakistan can play a crucial role in eradication of various such diseases which can wreak havoc on our fragile healthcare system. Educating masses about sanitation, proper health hygiene habits and disposal of waste can greatly subside the emergence of infectious enteric diseases in Pakistan.

## Ethical approval

None required.

## Sources of funding

Not funded.

## Author contribution

There's only one author.

## Registration of research studies

1. Name of the registry:
2. Unique Identifying number or registration ID:
3. Hyperlink to your specific registration (must be publicly accessible and will be checked).

## Guarantor

Rida Siddiqui.

## Provenance and peer review

Not commissioned, externally peer reviewed.

## Consent

None required.

## Declaration of competing interest

None to declare.

## References

[1] https://www.cdc.gov/cholera/infection-sources.html#:%7E:text=Cholera%20is%20an%20acute%20intestinal,cholera%20cases%20can%20be%20fatal.

[2] https://www.who.int/news-room/fact-sheets/detail/cholera#:%7E:text=Cholera%20is%20an%20extremely%20virulent,Most%20people%20infected%20with%20V.

[3] https://amp.cnn.com/cnn/2022/05/17/asia/pakistan-cholera-heatwave-climate-india-intl-hnk/index.html.

[4] https://www.thenews.com.pk/print/953609-129-confirmed-cholera-cases-in-karachi-in-2022-but-no-outbreak-say-health-officials.

[5] Jutla A., Whitcombe E., Hasan N., Haley B., Akanda A., Huq A. (2013 Sep). Environmental factors influencing epidemic cholera. Am. J. Trop. Med. Hyg..

[6] https://www.thethirdpole.net/en/pollution/91-of-karachis-water-unfit-to-drink.

